# Chemical Nature of Malaria

**Published:** 1871-12

**Authors:** D. Vaughan


### DENTAL REGISTER.

Vol. XXV.] DECEMBER, 1871. [No. 12.

### Communications.

---

### CHEMICAL NATURE OF MALARIA.

BY D. VAUGHAN, A. M., M. D.

Read before the Meeting of the Cincinnati Academy of Medicine, Monday, November 20, 1871.

From the peculiar manner in which the physical organization of man is adapted to the normal condition of the material agencies to which he is exposed, he is occasionally doomed to suffer not only from evils implanted in the human frame, but also from the great operations and changes transpiring in the external world. While the blessings of health are liberally dispensed in many parts of our globe, there are certain regions in which the afflictions of disease are felt with the greatest severity, and where they form the chief portion of the calamities which the human race have to endure. Though the unfavorable degree of heat or cold prevailing in many lands, or the great changes to which temperature is subject, may be detrimental to health, yet in numerous cases disease depends on causes of a more obscure character. At the close of the last century, when discoveries in pneumatic chemistry had given a vast extension to the domain of positive science, it was expected that an analysis of the air in unhealthy districts would reveal the cause of their insalubrity. It was soon found, however, that these hopes could not be realized by the labor of subsequent experimenters. No connection could be traced between the prevalence of diseases and the complement of oxygen, nitrogen or carbonic acid in the air; and the early methods of endiometry devised for finding the proportion in which these gases float around us were soon abandoned as uncertain and useless for indicating the great dangers which threaten the integrity of the human frame.

Although some doubts may exist in regard to the endemic character of many diseases, the connection of one class with certain localities seems to have been well established by all modern observation and experience. It is well known that the destructive inroads of intermittent fever are chiefly confined to those districts where the land is always impregnated with a superfluity of water, and rendered capable of filling the surrounding air with aqueous vapor. The resulting evils assume a more deleterious character in the marshes of warm climates, and their great severity is manifested when the fall of rain increases the amount of moisture in the land and in the air. As these moist localities are almost invariably the seats of a luxuriant vegetation, it has been generally supposed that their unhealthy condition arises from the decay of vegetable matter, which is produced so abundantly during summer. Carbonic acid, carbureted hydrogen, and other gases resulting from decay and putrefaction, have been frequently regarded as identical with the poisonous principle of marsh exhalations. Yet this opinion is rejected by more careful observers, as it is known that where these gaseous bodies are evolved in the greatest abundance their influence on the human frame, though often very injurious to the health, is not capable of developing any symptoms or affections analogous to those of intermittent fever.

But the more recent improvements in chemical analyses have served to show that the atmosphere has a more diversified composition than was first assigned to it by Lavoisier and his contemporaries. About thirty years ago Saussure found that when air from which the carbonic acid was removed was detonated with hydrogen, a small portion of carbonic acid was produced, thus evidently indicating that there must be carbon in some other form in the atmosphere. It was subsequently found by Boussingault that when perfectly dry air is passed over red hot oxide of copper a small amount of water is always generated. From these two experiments it was first inferred that carbon exists in the air, not only as carbonic acid, but also in the form of carbureted hydrogen. But this conclusion was not warranted by the experiments, as the result attending them would be caused by the presence of most kinds of organic matter. The existence of such matter in the air was subsequently rendered evident by the change which nitrate of silver sustains from long exposure. But a more satisfactory means of settling the question has been recently afforded by the use of permanganate of potash, and Dr. Angus Smith, of England, employing this reagent for the estimation of aerial organic matter, has found, among other important results, that an excessive quantity is contained over stagnant pools of water.

Though it may be impossible to ascertain by a direct analysis the various kinds of animal and vegetable matter which float in minute quantities in the atmosphere, yet some information on the subject may be obtained by examining the different sources from which this matter is supplied. As there is a far greater portion of carbon embodied in the vegetable than in the animal kingdom, it is on the changes attending vegetation that the amount of carbonaceous materials in the atmosphere mainly depend.

We are generally taught to look upon plants as mere fountains of oxygen gas, and to regard their vital functions as the great agency for renovating the air which has been rendered impure by the respiration of animals. But a closer examination of Nature will compel us to modify this pleasing picture which has been given of her operations by a theory which is not wholly as correct as it is beautiful. Without attaching any credit to the extravagant stories of the Upas tree, whose poisonous exhalations are said to be fatal to life for many miles around, there are many facts to show that it is not only during decay, but even in the course of vigorous vegetation, that plants add to the impurities of the air and increase its insalubrity.

Of all vegetable productions, the alkaloids, with few exceptions, hold a prominent place among the most deadly poisons known to the chemist. But in the very limited number of plants which produce them, these poisonous substances are found only in a small quantity; few of them are volatile, and there seems to be no possibility of their mingling with our atmosphere in a proportion which might be injurious or dangerous to the human frame. From the vegetable acids there is still less to be apprehended, as they are for the most part less deleterious to life, and as their great solubility in water is sufficient to remove them from the air. But the organic compounds which flow more rapidly into our aerial ocean, and at the same time partake most of the characters ascribed to malarious emanations, are the volatile or essential oils which are constantly emitted by living and decaying plants. From the emission of these volatile principles generally arises the peculiar smell or the perfume which the various parts of plants diffuse. Their presence in summer produces the pleasing or occasionally the disagreeable odors of our fields and forests; and the delightful fragrance of the groves of tropical climates give indications of the abundance in which the vapors of these oils are poured into the atmosphere.

That this class of bodies form an important part of the malarious emanations of marshes may be inferred from their ready vaporization and from the manner in which it is influenced by heat. Though most of the essential oils have their boiling points ranging between 300 and 450 degrees Fahrenheit, they emit an appreciable amount of vapor at extremely low temperatures. It may appear singular that liquids so reluctant to boil should be so volatile or so ready to evaporate; but this apparent anomaly is the necessary consequence of the great density of their vapors, and they observe a conformity to Dalton's law in regard to vaporization. Thus, for instance, of spirits of turpentine, which boils at 316 degrees Fahrenheit, the vapor would have at 100 degrees the same tension which that of water has at 4 degrees below zero, and this, according to the experiments of Gay Lussac, may be estimated at 1-600 of the atmospheric pressure. From this we may calculate by the law of Mariotte that the vapor would be 600 times more attenuated than it is at the boiling point, when the density is 4.76, or nearly five times that of air. From this it may be estimated that at the temperature of 100 degrees the air is capable of permanently holding in a state of diffusion 1-125 of its weight of the vapor of spirits of turpentine.

Without insisting on the absolute universality of Dalton's law, I am convinced that it will apply without any material error to the vaporization of the essential oils. As in the case of evaporated water, it is the power of heat alone that determines the amount of these bodies which can exist as vapor in a given volume of air or in a given space. At the temperature of 75 degrees, 500 parts of air would be capable of holding in this manner two parts of the vapor of spirits of turpentine, but it could retain only one part if the temperature were reduced to 50 degrees. As most of the volatile oils have a much higher boiling point than the one I have selected for illustration, they can pervade the atmosphere in a more attenuated condition. It would require about 2,000 parts of air at the temperature of 75 degrees, or 4,000 at 50 degrees, to hold in a state of vapor one part of the oil of Spirca, which boils at 383 degrees Fahrenheit. Generally, the ferment oils, and others which contain oxygen, have the highest boiling points, and require a higher temperature to maintain a given portion of them in a state of vapor above the earth's surface.

From the very limited amount of these bodies that can float in our aerial ocean, and the influence of heat on the existence of their vapors, there can be little doubt that in the marshes of warm climates, the great luxuriance of vegetation and also its decay would cause the air to be saturated with these volatile oils during the day, and that they would be deposited at night like dew, but in a less perceptible quantity. As evidence of the dependence of intermittent fevers on such exhalations, I may refer to the circumstance that in the most unhealthy districts where it prevails malaria assumes an increased malignity during the night. The fact seems to have much engaged the attention of medical writers, and Elliotson concludes that at night the malarious poisons must have been deposited from the air. That in the absence of any volatile or aerial poison mere changes of temperature might be productive of such distempers can not be admitted, as these changes are far greater in the dry and colder localities, which are comparatively free from intermittent fevers. From the researches of Tyndall, it appears that not only clouds, but even moist air, impede the radiation of heat, and, by thus preventing the earth from receiving too much heat during the day, and parting with much at night, they tend to avert great fluctuations in the thermal condition of climates.

The warmest regions of the earth produce most abundantly the volatile oils, as well as other organic compounds containing little or no oxygen, as if the separation of this element from carbon and hydrogen required all the energy which heat confers on vegetative power. As water contributes to the luxuriance and vigor of vegetation, it must also increase the production of these volatile bodies, and send them more rapidly into the air by a kind of exhalation peculiar to vegetable life. The agency of this fluid on dead organic matter is attended with similar effects. The essential oil of (spirea) and that of bitter almonds are indebted to aqueous action for their origin, and generally, in accelerating decay and putrefaction, water is the means of introducing numerous ferment oils into the atmosphere. But it is to the slight solubility of these volatile bodies in water that we must ascribe much of the evil effects with which rains, and especially occasional showers, are attended in malarious districts. The volatile oils which rise high above the ground are brought down by the descending drops of rain, and the water thus impregnated with them becoming warm, they are again exhaled from the soil; but they are rendered more injurious to man by being thus confined to the lower stratum of the atmosphere and diffusing themselves throughout its mass.

The foregoing principles will serve to explain some facts which are at variance with the received theory of intermittent fevers. These diseases do not withhold their attacks until the work of decay proceeds on any considerable scale; but they commence and often display their greatest malignity when vegetation is in its greatest vigor, thus showing that they are at least as much dependent on the exhalations of living as of dead plants. In many cases, also, their attacks begin so soon after rain that they can not depend on the results of putrefaction alone. The insalubrity of marshes appears to have some dependence on the kinds of vegetation which they produce; as, for instance, heat formations, though always in a moist condition, are free from malaria, and the exemption is principally due to the absence of plants capable of affording the noxious volatile oils during their growth and decay.

Apart from the effects which I have ascribed to occasional showers of rain, the great density of the vapors of the essential oils would tend to keep them close to the earth's surface, and thus render them more capable of affecting the human frame. Even in the absence of chemical union, unequally dense gases would mix with uniformity; but to the law of diffusion observed in these results in gaseous mixtures, vapors do not strictly conform; and when their tension and expansibility are feeble, their position is determined by their specific gravities—the heaviest sinking to the lowest stratum of air. A like deviation from the principle of mutual diffusibility seems to prevail in gases when highly rarified, as the upper part of the sun's atmosphere consists mainly of hydrogen gas, the lightest body known. It has, however, been proved by Faraday's experiments that heavy vapors are incapable of rising above a certain level, so that the vapors of the volatile oils must be confined to the lower stratum of the atmosphere, and thus rendered more injurious to man.

But the most conclusive evidence on this question is afforded when the inevitable changes which these emanations must undergo is taken in connection with the peculiar manner in which malaria is known to decline. From observations made in Italy it has been found that the virulence of marsh poison is manifested not more than three miles from its source. During the short time of its diffusion within this range it loses its noxious properties, and there is no class of vegetable productions which change so rapidly as the essential oils, especially after their vaporization. If exposed to air in a liquid form, they gradually become resins by combining with oxygen; but when blended with the atmosphere in a state of vapor, their oxidation and change to resins must take place with far greater rapidity. It is the oxidation of the volatile oils which some flowers emit that gives them a temperature higher than that of other parts of the plant; but in their tendency to oxidize these bodies exhibit much difference, though not to the same extent as the fixed oils.

It may be necessary, however, to extend our inquiries to a consideration of the manner in which the human frame could be so seriously affected by vapors or exhalations from which little danger might be apprehended. Oxidation converts the volatile oils into resinous bodies, many of which are used in the art as varnishes, and are adapted for the purpose chiefly on account of their insolubility in water. The destructive effect of the vapor of turpentine to insects may be ascribed to the resinous coating which, by oxidation, it forms on their bodies, as this would impede, to a fatal extent, the function of respiration, which in this class of animals is chiefly performed through the skin. But as in man there is between thirty and sixty times more carbonic acid exhaled from the lungs than from the skin, the same fatal effects could not be reasonably expected to follow from the entire suppression of cutaneous respiration, even though essential oils were abundant in the air to produce it by giving the body an impervious coating of resinous matter. A widely different opinion has been drawn from the experiments of Becquerel and Breschel, who found that on varnishing rabbits, after shaving off their hair, these animals rapidly declined in temperature, and died in a short time. But it does not seem legitimate to attribute the fatal effects exclusively to the suppression of cutaneous respiration, as there is reason to believe that the impervious coating might obstruct or interfere with the functions of the nervous fibers in the vicinity of the surface of the body, and in this way seriously affect calorific and vital action.

In our inquiries respecting the origin of animal heat, we must admit, with the chemist, that in every department of nature the creation of heat is impossible, and that the chemical changes transpiring in the human system are adequate to supply the amount of it necessary for the maintenance of life. We can not, however, ignore the results of those important physiological experiments which prove the dependence of vital heat on nervous action and the integrity of the nervous tissues. From the several facts which have been adduced to support the two rival theories on this subject, it seems reasonable to conclude that animal heat is the offspring of a chemical action which the blood undergoes, but which the nerves instigate and maintain by supplying it with matter more disposed to oxidize. That this property is very prominent in nervous and cerebral matter, is known from the result of experiments. As a small quantity of dry wood is so often the means of kindling the largest coal fires; as a little yeast, or some other nitrogenous matter, brings into fermentation a large amount of dissolved sugar; as the minute portion of the fulminating compound in cartridges inflames the gunpowder in modern firearms and makes them deal destruction among contending armies, so the little matter given by the nerves originate and keep up the oxidation of the blood. As this must be attended with a caloric influence, the nerves are thus instrumental in producing an amount of heat perhaps several hundred times greater than could arise from any chemical action in the matter inclosed in their tubes or tissues.

From some recent experiments and researches of Frankland, it appears that the muscles are the seats of the combination of the blood with the oxygen taken in at the lungs. My own investigations have, indeed, led me to a similar conclusion, but they show, also, that oxidation is most active at the point where the fibers of the motor nerves terminate in the muscles. Now, if, through the medium of endosmosis, cutaneous absorption or the aeration and circulation of the blood, any essential oil were introduced into the muscles, their oxidation about the entrance of the nerves would form resins, which, instead of being removed from the system, would only serve to obstruct or to seal up the nervous tubes, and thus diminish or suspend caloric action. In most cases, however, these barriers or restraints would be removed by the pressure of matter accumulated in the nerves or by some vital reaction, an excessive oxidation of the blood and development of heat would then take place, but the resinous matter would still remain, and with its aid the volatile oils impregnating the system would require only a few days to obstruct again or to stop the communications between the nerves and muscles.

In this manner we may account for the chill with which intermittent fever commences, the subsequent heat and sweating, and the recurrence of the paroxysm every few days. As some of the ferment oils have the character of alcohols, they would not act in the precise way I have described, but they might prove injurious to the system by dissolving the fatty matter, and, on losing their solvent power by oxidation, depositing it at the junction of nervous and muscular fibers. When, in consequence of the debility of the human frame, or the great abundance of vegetable poisons, the fatty or resinous obstructions can not be removed, remittent fever may result, either from some rupture of the nervous tissue, or from the imperfect manner in which the blood is oxidized.

But the existence of the latter class of diseases, or at least their malignity, seems to depend on poisons generated in the human frame, and it is perhaps to changes in animal matter that we are to look for the origin of those poisons and miasms which have the most noxious characters, which retain them for the longest time and diffuse them over the widest range, as they are transported to distant localities by the flow of rivers or by those great currents of air which are in perpetual motion over the surface of our planet.

